# Health Tract, No. 256

**Published:** 1866-10

**Authors:** 


### ACCIDENTS.

Many of the so-called accidents of life by which health is irrecoverably lost, if indeed not more immediately fatal, are the legitimate result of ignorance, indifference, or thoughtlessness and which with a very little reflection might have been avoided. Nothing is more common than for girls and women to put a pin in their mouths, intending it to be but for an instant, and yet forgetting it, on a sudden impulse to laughter, it is drawn into the throat,or lungs themselves, requiring a delicate, painfnl and dangerous surgical operation in order to extract it. Perhaps nine persons out of ten in passing through a wood or harvest field, will take a twig or a straw and put it into their mouth, and before they are aware, it has made its way downwards causing the most distressing cough or strangulation, sometimes inducing hemorrhage or convulsions. Recently a printer in Baltimore, after a violent fit of coughing expelled a brass nail an inch long, much corroded. It had been "accidentally" swallowed two years before, and for all that length of time had been a constant source of annoyance, bleedings and ill health. There is a peculiar tendency in children to put things to their nose or ears, and especially if a little rounded, a little slip propels them into the cavity. A girl, five year old, made a motion as if to smell a little black bean; it suddenly disappeared; a surgeon was sent for and probed the nostrils, but could feel nothing. Six months later, it was blown from the nose; we saw it a few moments afterwards all shrivelled and wrinkled. Very recently a little fellow introduced a foreign body into his ear; as probing for it occasioned a great deal of pain, chloroform was used to facilitate the extraction, which failed, and at the same time the child was discovered to be dead. Children should be taught from infancy that life may be lost in ways above named, and that on no pretence whatever, for a single instant ought anything to be put into the mouth, which is not in the nature of food, and to keep hard objects away from the ears and nostrils.

Very lately a man went down into a well and was observed by his brother to dip his head forward and remain still and silent; the brother in haste went down to his assistance and was observed to make precisely the same motion, when a third brother went to his rescue with a similar result; they were all found to be dead from the suffocating gas at the bottom of the well. A bunch of straw, paper, or shavings should be thrown burning into a well before descending; if it goes out, death is there. The first brother might have been saved by throwing cold water upon him, to absorb the gases and carry more oxygen down.

